# Is heat pain detection threshold associated with the area of secondary hyperalgesia following brief thermal sensitization? A study of healthy volunteers - design and detailed plan of analysis

**DOI:** 10.1186/s12871-016-0193-2

**Published:** 2016-05-31

**Authors:** Morten Sejer Hansen, Jørn Wetterslev, Christian Bressen Pipper, Mohammad Sohail Asghar, Jørgen Berg Dahl

**Affiliations:** 1 Department of Anaesthesiology, 4231, Centre of Head and Orthopaedics, Rigshospitalet, Blegdamsvej 9, Copenhagen, 2100 Denmark; 2 Copenhagen Trial Unit, Centre for Clinical Intervention Research, dep. 7812, Blegdamsvej 9, Copenhagen, 2100 Denmark; 3 Section of Biostatistics, Faculty of Health, Copenhagen University, Øster Farigmagsgade 5, Copenhagen, 1014 Denmark; 4 Department of Anaesthesiology, dep. Z, Bispebjerg Hospital, Bispebjerg Bakke 23, Copenhagen, 2400 Denmark

**Keywords:** Pain, Anaesthesiology, Physiology, Hyperalgesia, Central sensitization, Quantitative sensory testing

## Abstract

**Background:**

Several factors are believed to influence the development and experience of pain.

Human clinical pain models are central tools, in the investigation of basic physiologic pain responses, and can be applied in patients as well as in healthy volunteers. Each clinical pain model investigates different aspects of the human pain response.

Brief thermal sensitization induces a mild burn injury, resulting in development of primary hyperalgesia at the site of stimulation, and secondary hyperalgesia surrounding the site of stimulation. Central sensitization is believed to play an important role in the development of secondary hyperalgesia; however, a possible association of secondary hyperalgesia following brief thermal sensitization and other heat pain models remains unknown. Our aim with this study is to investigate how close the heat pain detection threshold is associated with the size of the area of secondary hyperalgesia induced by the clinical heat pain model: Brief thermal sensitization.

**Methods and design:**

We aim to include 120 healthy participants. The participants will be tested on two separate study days with the following procedures: i) Brief thermal sensitization, ii) heat pain detection threshold and iii) pain during thermal stimulation. Additionally, the participants will be tested with the Pain Catastrophizing Scale and Hospital Anxiety and Depression Scale questionnaires.

We conducted statistical simulations based on data from our previous study, to estimate an empirical power of 99.9 % with α of 0.05. We define that an R^2^ < 0.25 and predictive intervals larger than +/−150 cm^2^ are indications of a weak association.

**Discussion:**

The area of secondary hyperalgesia may serve as a quantitative measure of the central sensitization induced by cutaneous heat stimulation, and thus may be a biomarker of an individual’s pain sensitivity. The number of studies investigating secondary hyperalgesia is growing; however basic knowledge of the physiologic aspects of secondary hyperalgesia in humans is still incomplete. We therefore find it interesting to investigate if HPDT, a known quantitative sensory test, is associated with areas of secondary hyperalgesia following brief thermal sensitization

**Trial registration:**

Clinicaltrials.gov (Identifier: NCT02527395). Danish Research Ethics Committee (Identifier: H-8-2014-012). Danish Data Protection Agency (Identifier: 30-1436).

**Electronic supplementary material:**

The online version of this article (doi:10.1186/s12871-016-0193-2) contains supplementary material, which is available to authorized users.

## Background

Psychology, physiology, ethnicity, sex and psychosocial background are just some of the factors believed to contribute to the pain experience in the individual. However, the knowledge of the individual mechanisms responsible for the development of acute and chronic pain is still incomplete [1].

Human clinical pain models are central tools, in the investigation of basic physiologic pain responses, and can be applied in patients as well as in healthy volunteers [2, 3].

With the pain model brief thermal sensitization (BTS), the skin is heated to 45 °C for 3 min. resulting in the development of primary hyperalgesia at the site of stimulation, and secondary hyperalgesia surrounding the site of stimulation [4–8]. The area of secondary hyperalgesia is characterised by reduced thresholds for mechanical stimulation, and the size of the area can be quantified by monofilament stimulation. Current evidence indicates that the development of secondary hyperalgesia to punctate mechanical stimuli following a standardised injury is caused by central changes in response to a conditioning stimulus and transmitted by A-delta fibers [1, 9–11]. The secondary hyperalgesia elicited by a clinical pain model may thus be a result of central sensitization. Central sensitization reflects a functional change in the neuron properties by increased synaptic efficacy and membrane excitability as well as decreased synaptic inhibition [1, 9]. This functional change in the neurons and nociceptive pathways results in pain threshold reduction, increased response to noxious stimulation, and prolonged duration of pain following noxious stimulation [1, 9]. Central sensitization encompasses both an early and late phase, with the early phase independent of transcription and the late long-lasting phase transcription dependent [9]. The transcription dependent long lasting phase of central sensitization is believed to play an important role in several different pain conditions and syndromes, e.g. osteoarthritis and fibromyalgia [1, 9, 12, 13].

Secondary hyperalgesia following clinical pain models has been demonstrated to be a robust phenomenon, and can be applied when investigating basic pain physiology in humans [4–8, 14–30].

Recently, a meta-analysis [24] of 10 studies (9 published) [4–6, 8, 19, 21, 23, 26, 30] indicated that healthy volunteers had; (I), a large inter-individual variation with regard to the magnitude of the area of secondary hyperalgesia after exposure to identical pain stimuli. (II), that areas of secondary hyperalgesia may be an identifiable phenotypic indicator and that (III), they may be predictive of individual pain responses. Furthermore, in a recently published study [31], results have indicated that increasing heat pain detection threshold (HPDT) may be associated with decreasing size of the area of secondary hyperalgesia. However, the study indicates that HPDT only offers a very modest explanation of the variation in the area of secondary hyperalgesia following standardised burn injury, suggesting that HPDT and secondary hyperalgesia may be two different pain entities.

Moreover, one recent study found indications of structural cerebral differences and differences in neuronal activation to noxious stimulation, in healthy volunteers with a large vs. small area of secondary hyperalgesia following a first degree burn injury [25]. Finally, the study also found suggestions of an association between large areas of secondary hyperalgesia and high anxiety scores.

The different physiologic properties of BTS and HPDT [1, 9–11, 32] suggests that they may be two different pain entities; however the current evidence on the basic physiologic properties of the areas of secondary hyperalgesia in humans remains incomplete. The predictive value of HPDT on postoperative pain [33–35], suggests that HPDT may partly provide insight on an individual’s pain sensitivity; however, our previous results indicate that HPDT and the development of secondary hyperalgesia may only be weakly associated. This result challenges the current assumption that areas of secondary hyperalgesia may be a marker for an individual’s central sensitization and ultimately the pain sensitivity. This rather surprising result needs to be explored further. A thorough evaluation of the basic physiologic properties in the development of secondary hyperalgesia following BTS is necessary in order to exploit the full potential of this clinical pain model, and may provide novel insight in the development of acute and chronic pain.

In this prospective cohort study we therefore aim to investigate how close the HPDT, evaluated on two separate study days, is associated with the size of the area of secondary hyperalgesia induced by BTS. We hypothesise that HPDT and area of secondary hyperalgesia are two predominantly independent entities, and thus, are weakly associated.

## Methods/design

The method and design of this study is based upon a previous study by our own research group [31]. The recruiting of study participants and commencement of the study was initiated on October 1, 2015.

### Study participants

Only healthy male volunteers will be included in the study. The investigator will provide the study participants with written and oral information about the study, as well as information concerning possible risk and side effects. The study participants will sign a written informed consent, and will receive EUR 20 (USD 27) per hour for their participation in the study. Inclusion and exclusion criteria are presented in Table 1.

**Table 1:** Inclusion and exclusion criteria

Inclusion criteria	Exclusion criteria
Age ≥18 years and ≤35 years	Study participants who cannot cooperate to the test.
Speak and understand the Danish language	Study participants who have a weekly intake of >21 units of alcohol, or a have consumed >3 units of alcohol within 24 h before study day.
Healthy male	Study participants with a substance abuse, assessed by the investigator.
Signed informed consent	Study participants, who have consumed analgesics less than 3 days before study day.
	Study participants, who have consumed antihistamines less than 48 h before study day.
	Study participants who have consumed antidepressant medication during the last 30 days before the study
	Study participants who have consumed prescription medicine during the last 30 days before the study.
	Study participants with neurological illnesses.
	Study participants with chronic pain
	Study participants with psychiatric diagnoses
	Study participants with tattoos on the extremities
	Study participants with eczema, wounds or sunburns on the sites of stimulation.
	Study participants with a Body Mass Index of >30 kg/m^2^ or <18 kg/m^2^

### Setting

The study is conducted in a quiet secluded room (temperature: 22–25 °C), where only the study participant and the investigator will be present. The study participants will be in a supine position, lying on their back, during the assessments. The study will be conducted at the Department of Anaesthesiology, 4231, Rigshospitalet, Copenhagen, Denmark.

### Study design

The study is a confirmatory study designed to investigate the association between area of secondary hyperalgesia and HPDT. The study consists of a screening/information day and two study days. To prevent carry-over effects of the applied tests, the screening day and each of the two study days must be separated with a minimum of seven days. The aim with our study is to investigate the association between HPDT and area of secondary hyperalgesia, thus the order of the stimulations (HPDT and BTS) is randomised for each participant for each study day. Pain during 1 min. thermal stimulation (p-TS) is conducted subsequent to BTS and HPDT. Likewise, in order to adjust for possible carry-over effects between HPDT and BTS we conduct experimental pain testing on two separate study days with the experimental pain testing sequence randomised for each study day (see Randomisation, allocation concealment and blinding).

The study participants will thus be tested with three types of clinical pain models on two separate study days. The following clinical pain models will be applied: Brief thermal sensitization (BTS), heat pain detection threshold (HPDT) and pain during thermal stimulation (p-TS) (see Assessments and tests). Furthermore, the study participants will complete the two psychological tests, Pain Catastrophizing Scale (PCS) and Hospital Anxiety and Depression Scale (HADS) (see Assessments and tests).

The screening day and the two identical study days will proceed as follows:

Screening day:

- Oral information regarding the study is delivered by the primary investigator. Informed consent and written authority will be handed out for the participant to sign. The participant will be given time to consider the information.
- Short medical history by the primary investigator. Thorough screening of in- and exclusion criteria. Measurement of height, weight, blood pressure and pulse.
- In order to familiarise the study participant with the types of pain conditioning, the 3 types of pain conditioning test are presented and performed on the study participant.
- Distribution of PCS and HADS (in Danish), together with an opaque envelope, in which the answered tests are to be put, and a sealed envelope will be returned to the primary investigator on the first study day.
- Potentially clarifying questions concerning the study or PCS and HADS are answered.

Study day 1 and 2:

- Inspection of signed informed consent and written authority.
- Study day 1: Hand in of PCS and HADS in an opaque sealed envelope. The envelope is first opened when all study participants have completed study day 1 and 2.
- The study participant is placed on a bed in the supine position.
- The timeline of tests performed will be as follows:
  - ○ Commencement of study 0 min: BTS or HPDT (depending on randomisation)
  - ○ At 7 min: BTS or HPDT (depending on randomisation)
  - ○ At 10 min: p-TS

### Assessment and tests

#### Brief thermal sensitization (BTS)

A computer-controlled thermode (Somedic MSA Thermotester™; size 2.5 × 5 cm) is placed on the participant’s skin. The initial temperature of the thermode is 32 °C, and temperature is increased 1 °C/s until it reaches 45 °C. The temperature of the thermode remains 45 °C for 3 min., and while the thermode still has contact with the skin, and has a temperature of 45 °C, the assessment of secondary hyperalgesia is conducted. The assessment of secondary hyperalgesia will approximately take 1–2 min, with a maximum duration of heat stimulation of 5 min. The test is conducted centrally on the anterior part of the right thigh.

#### Assessment of secondary hyperalgesia

The area of secondary hyperalgesia is quantified after stimulation with a 19G monofilament (von Frey hair) in 4 linear paths arranged in 90° around the centre of stimulation. Stimulation will begin 15 cm. from the centre of stimulation and advance in steps of 5 mm. with 1 s intervals towards the centre of stimulation. When the participant states a clear change in sensation (intense pricking, burning, tenderness) the place will be marked with a felt pen and the transverse and longitudinal axes will be measured with a pliable measuring tape for later rectangular area calculation.

#### Heat pain detection threshold (HPDT)

Heat pain detection threshold represents the lowest temperature that is perceived as painful, when heating the skin with the computer-controlled thermode. The initial temperature is 32 °C, and temperature is increased 1 °C/s. The participant is asked to press a button when the heat is perceived as painful. If 52 °C (the maximum temperature for the thermode) is reached before the participant’s threshold has been registered, the thermode will automatically return to the initial temperature of 32 °C to prevent excessive tissue damage. The HPDT is calculated as an average of four stimulations. Each stimulation will be performed with an interval of 6–10 s. The test is conducted centrally on the anterior part of the dominant lower arm.

#### Pain during thermal stimulation (p-TS)

The computer-controlled thermode is placed on the participant’s skin. The initial temperature of the thermode is 32 °C, and temperature is increased 1 °C/s until it reaches 45 °C, where it remains for 1 min. During this the participant evaluates the pain with an electronic VAS-scale. The participant will continuously evaluate the pain using the electronic VAS-scale because of the fluctuations in pain intensity during this type of stimulation. The equipment (Somedic USB-VAS) automatically calculates a VAS-score under the curve (VAS-AUC) and a maximum VAS-score for the time period. The participant will not be able to see the computer screen during the measurement, and each pain evaluation will be independent of the previous evaluation. The test is conducted centrally on the anterior part of the non-dominant lower arm.

#### Pain assessment

Visual analogue scale (VAS), index from 0–100 mm, where 0 mm represents “no pain”, and 100 mm represents “the worst pain imaginable”.

#### Hospital Anxiety and Depression Scale (HADS)

HADS is a questionnaire consisting of 14 questions, and is a four-point Likert scale with values from 0–3. HADS can be subdivided into HADS-A, evaluating anxiety, and HADS-D, evaluating depression. The highest achievable score is 42, and a total HADS-score will estimate the participant’s level of distress. To evaluate anxiety and depression separately, HADS-A and HADS-D must be evaluated separately, with a maximum score of 21 in the two subtests.

The interpretation of the score in HADS-A and HADS-D is as follows:

- 0–7: Normal
- 8: Mild level of anxiety/depression
- 11–15: Moderate level of anxiety/depression
- ≥16: Severe level of anxiety/depression

The HADS questionnaire is to be completed before study day 1.

#### Pain Catastrophizing Scale (PCS)

PCS is a questionnaire consisting of 13 questions. PCS is a five-point Likert scale with values from 0–4, and can be subdivided into 3 subtests, that each evaluates the central elements in catastrophizing: Rumination, magnification and helplessness. The highest achievable score is 52, and with separate evaluation of the three subtests, the three different elements can be assessed individually. To evaluate the three elements separately the 13 questions must be evaluated in the three following subgroups:

- Rumination: The sum of question 8, 9, 10, 11. Maximal sum =16
- Magnification: The sum of question 6, 7, 13. Maximal sum = 12
- Helplessness: The sum of question 1, 2, 3, 4, 5, 12. Maximal sum = 24

The PCS questionnaire is to be completed before study day 1.

### Randomisation, allocation concealment and blinding

The three types of clinical pain models consist of BTS, HPDT, and p-TS (see Assessments and tests). p-TS are conducted subsequent to BTS and HPDT. The order of BTS and HPDT is randomised, by a random allocation sequence, computer-generated by Copenhagen Trial Unit and stored in sealed and opaque envelopes to secure adequate allocation concealment.

Completed psychological tests will be kept in opaque sealed envelopes. The blinding will be broken when all volunteers have completed the two study days.

### Outcomes

#### Primary outcome

How close is the heat pain detection threshold associated with the size of the area of secondary hyperalgesia induced by BTS? The association will be expressed in R^2^ and with prediction intervals for the area of BTS given fixed values of HPDT.

#### Secondary outcomes

To investigate:

1. How close the VAS-AUC following p-TS is associated with the size of the area of secondary hyperalgesia induced by BTS.
2. How close the Max VAS-score following p-TS is associated with the size of the area of secondary hyperalgesia induced by BTS.
3. How close the score of PCS is associated with the size of the area of secondary hyperalgesia induced by BTS.
4. How close the score of HADS is associated with the size of the area of secondary hyperalgesia induced by BTS.
5. How close the subscales in the two psychological tests (PCS-rumination, PCS-magnification, PCS-helplessness, and HADS-Anxiety, HADS-Depression) are associated with the size of the area of secondary hyperalgesia.

### Hypothesis

Our hypothesis is that HPDT and area of secondary hyperalgesia elicited by BTS are two predominantly independent entities, and that the area of secondary hyperalgesia is poorly explained by HPDT. We will take R^2^ < 0.25 and predictive intervals larger than +/−150 cm^2^ as indications of a weak association.

### Simulation based assessment of sample size

Simulation based power calculations are based on data from our own previous study [31]. In particular power calculations are based on a linear relationship between BTS and HPDT derived from inference of data from this previous study.

With α of 0.05 and 120 participants we estimated an empirical power of over 99.9 % using simulations. This provides adequate power to confirm if heat pain detection threshold and area of secondary hyperalgesia following BTS are to large extent independent entities, and thus weakly associated.

Please see the online available Additional file 1 (intrep3-ver3-2015) for the full simulation study.

### Data analysis plan

#### Plan of statistical analysis of primary outcome

The association of HPDT with the area of secondary hyperalgesia will be investigated by linear regression on the estimated best linear unbiased predictors (EBLUPS) of the individual area of secondary hyperalgesia. The individual area of secondary hyperalgesia will be adjusted with the body surface area of the individual participant. Significance of the predictor will be assessed by ANOVA methods. The ability of the HPDT to predict individual variations in areas of secondary hyperalgesia will be investigated by linear regression on the EBLUPS of the individual area of secondary hyperalgesia. Significance of the predictor will be assessed by ANOVA methods and the predictive ability will be quantified by summary of prediction error including 95 % prediction interval.

Relevant examples will be presented illustrating how accurate HPDT predicts secondary hyperalgesia in individual patients or vice versa.

Subgroup analyses based will be performed if at least 15% of the study population differs in ethnicity.

#### Plan of statistical analysis of secondary outcomes

For each of the 5 secondary outcomes the association of VAS-AUC (following p-TS), Max VAS-score (following p-TS), PCS, HADS, and subscales of PCS and HADS with the area of secondary hyperalgesia will be investigated by linear regression on the estimated best linear unbiased predictors (EBLUPS) of the individual area of secondary hyperalgesia. Significance of the predictor will be assessed by ANOVA methods. In case of significance, the ability of the five variables to predict individual variations in areas of secondary hyperalgesia will be investigated by linear regression on the EBLUPS of the individual area of secondary hyperalgesia. Significance of the predictors will be assessed by ANOVA methods and their predictive abilities will be quantified by various summaries of prediction errors including 95 % prediction intervals.

Relevant examples will be presented illustrating how accurate the five variables predict secondary hyperalgesia in individual participants or vice versa.

### Missing data

For all analyses, an intention-to-test (ITT) analysis will be performed including all subjects that participated in the first study day. Analyses will be based on all observed data. If missingness exceeds 5 % and there is indication of violation of “missing completely at random” (MCAR) by a statistical significant Littles test, a sensitivity analysis based on an appropriate model for missingness at random (MAR) or not at random (MNAR) will be performed.

### Statistical significance

All *p*-values are evaluated at a 5 % significance level. *P*-values for multiple comparisons are adjusted for multiple testing by means of single-step correction [36].

### Software

All analyses will be made using the open source statistical programming environment R [37].

### Ethics

The applied methods do not cause damage to the skin or have any other long-term adverse effects. In rare cases, changes comparable with a first degree sun burn may appear.

The study will be conducted in accordance with the principles of the Declaration of Helsinki. Written informed consent will be obtained from all the study participants. The participants will be aware of the rare possibility of a first degree burn prior to consenting.

The protocol is approved by the local Danish Research Ethics Committees (Identifier: H-8-2014-012) and the Danish Data Protection Agency (Identifier: 30-1436). The study is also reported on the international database clinicaltrials.gov (Identifier: NCT02527395).

## Discussion

In the present study we aim to investigate how close the HPDT is associated with the size of the area of secondary hyperalgesia induced by BTS. Based on a previous study [31], we hypothesise that HPDT and area of secondary hyperalgesia elicited by BTS are two predominantly independent entities, and that the area of secondary hyperalgesia is poorly explained by HPDT. We estimate that an R^2^ < 0.25 and predictive intervals larger than +/−150 cm^2^ are indications of a weak association. Because we hypothesise that HPDT and the area of secondary hyperalgesia following BTS are possibly two different pain entities we need a high statistical power to ensure that our result is valid. We conducted statistical simulations with the raw data from our previous study [31], and have estimated that with 120 participants we achieve an empirical power of 99.9 %. With an α of 0.05 and an estimated empirical power of almost 100 % we feel confident that the risk of type 2 error is minimal.

The area of secondary hyperalgesia may serve as a quantitative measure of the central sensitization induced by cutaneous heat stimulation, and thus may be a biomarker of an individual’s pain sensitivity. The stimuli following HPDT and BTS are transmitted in A-delta and C-fibres [32].

With 1 °C/s. increase in temperature HPDT primarily activates C polymodale nociceptive afferents, and to a lesser degree A-delta fibres [32]. However, the development of secondary hyperalgesia to punctate mechanical stimuli is centrally mediated and occurs as a result of central plasticity, and has been demonstrated to involve nerve signals primarily in A-fiber nociceptors, not C-fibres [1, 9–11]. HPDT has been demonstrated to be highly reproducible, and may serve as a predictor of pain responses following surgery [33–35], but the current scientific evidence investigating the predictive value of areas of secondary hyperalgesia remains sparse. Thus, if this study demonstrates a weak association between HPDT and secondary hyperalgesia, it does not mean that secondary hyperalgesia as a biomarker for pain sensitivity is useless, just that HPDT and secondary hyperalgesia following BTS may be two different pain entities, and express different aspects of pain physiology. Studies investigating the value of secondary hyperalgesia in predicting postoperative pain are warranted in order to clarify this aspect.

Ethnicity, sex, obesity and hormone cycle are all factors that may influence pain sensitivity [20, 38–50]. In this study we aim to investigate the association between HPDT and secondary hyperalgesia following BTS, and in order to isolate a possible association between HPDT and secondary hyperalgesia we chose to include healthy male volunteers only. The inclusion of a homogenous male population enables us to focus on the association between HPDT and secondary hyperalgesia, instead of the influence of the individual participant characteristics. This may also be a limitation since the result of the study is primarily applicable in male volunteers. However, to our knowledge, this is the first study investigating the association between HPDT and the area of secondary hyperalgesia following a cutaneous heat injury, and, thus we need to limit known and unknown variables that may influence the primary outcome and distort the signal.

Several clinical studies have demonstrated associations between pain and psychological variables [51–54]; however, the influence of psychological variables on experimental pain still remains unclear [55]. In this study we also aim to investigate a possible association between secondary hyperalgesia and anxiety, depression and pain catastrophizing. One study by Salomon et al. [56] demonstrated that a cognitive-behavioral intervention reducing PCS may reduce the size of the area secondary hyperalgesia. Salomon et al. suggests that central sensitization can be modified by intervening and altering the pain-relating thoughts, and, thus suggests a possible association between the size of secondary hyperalgesia areas and PCS.

The number of studies investigating secondary hyperalgesia as a biomarker for central sensitization and pain sensitivity is growing; however basic knowledge on the physiologic aspects of secondary hyperalgesia in humans is still incomplete. We therefore find it interesting to investigate if HPDT, a known quantitative sensory test [57], is associated with areas of secondary hyperalgesia following BTS.

## Abbreviations

°C, Degrees celsius; ANOVA, Analysis of variance; AUC, Area under the curve; BTS, Brief thermal sensitization; Cm, Centimetres; EBLUPS, Estimated best linear unbiased predictors; EUR, Euro; HADS, Hospital anxiety and depression scale; HPDT, Heat pain detection threshold; MAR, Missing at random; MCAR, Missing completely at random; Min, minutes; mm, Millimetre; MNAR, Missing not at random; PCS, Pain catastrophizing scale; p-TS, Pain during 1 min. thermal stimulation; Sec, Seconds; USD, United States Dollars; VAS, Visual analog scale.

## Additional file

Additional file 1: Simulation study. (PDF 204 kb)
