# Supplementary material for: Is heat pain detection threshold associated with the area of secondary hyperalgesia following brief thermal sensitization? A study of healthy volunteers - design and detailed plan of analysis
Source: BMC Anesthesiol. 2016 May 31;16:28. doi: 10.1186/s12871-016-0193-2 (PMC4888470; doi:10.1186/s12871-016-0193-2)
Supplement: Additional file 1: — Simulation study. (PDF 204 kb) [file 12871_2016_193_MOESM1_ESM.pdf]

Department of Biostatistics,
University of Copenhagen

February 16, 2015
2015
CP

Internal report 3 version 2

A simulation study on the power to detect an effect of HPDT and VAS on BTS

Morten Sejer Hansen (RH)

Contents

1 Simulation design 1

2 Estimation and inference 1

3 Obtaining parameter values for the simulations 2

4 Results 3

5 Appendix: R code 4

Christian Pipper

Internal reports are preliminary working papers which are confidential unless an explicit agreement to the contrary has been reached between the clients and the author of the report.

1 Simulation design

We consider n persons. For the ith person on the we simulate individual BTS levels according to the model

$$BTS_i = \mu + \beta_1 \cdot HPDT_i + \beta_2 \cdot AVAS_i + \beta_3 \cdot MVAS_i + \varepsilon_i, \qquad (1)$$

where μ is the overall level of the response, $HPDT_i, MVAS_i, AVAS_i$ correspond to the eblups (individual summaries of HPDT, MVAS, AVAS) extracted as described in internal report 2 version 1, $\beta_1, \beta_2, \beta_3$ are effects of predictors, and ε_i are measurement errors distributed according to $N(0, \sigma^2)$.

Values of HPDT, MVAS and AVAS are sampled with replacement from the simultaneous empirical distribution obtained from the hyperalgesia 1 study.

We also consider the alternative model

$$BTS_i = \nu + \beta \cdot HPDT_i + \eta_i, \qquad (2)$$

where η_i are measurement errors distributed according to $N(0, \sigma_1^2)$.

All simulations are conducted in R version 3.0.2 (www.r-project.org)

2 Estimation and inference

For a given simulated data set according to (1) inference is based on multiple linear regression The effect of HPDT and the simultaneous effect of MVAS and AVAS is assessed by F-tests.

For a given simulated data set according to (2) inference is based on simple linear regression The effect of HPDT by a t-test.

All analyses are conducted with the R-functions `lm()` and `lmer()` available from the `lme4` add-on R package.

3 Obtaining parameter values for the simulations

We analysed measurements from the first two days of the hyperalgesia 1 data according to (1) and obtained the following parameter model output

```
Call:
lm(formula = eblups2 ~ hpdt2 + avas2 + mvas2, data = datatilsim)

Residuals:
     Min       1Q   Median       3Q      Max
-236.104  -69.970   -6.324   79.505  308.781

Coefficients:
              Estimate Std. Error t value Pr(>|t|)
(Intercept)  4.392e-12  1.671e+01   0.000  1.00000
hpdt2       -2.424e+01  8.423e+00  -2.878  0.00605 **
avas2        6.210e-02  5.260e-02   1.180  0.24388
mvas2       -8.432e-01  2.495e+00  -0.338  0.73693
---
Signif. codes:  0 ?***? 0.001 ?**? 0.01 ?*? 0.05 ?.? 0.1 ? ? 1

Residual standard error: 118.2 on 46 degrees of freedom
Multiple R-squared:  0.2536,Adjusted R-squared:  0.2049
F-statistic: 5.209 on 3 and 46 DF,  p-value: 0.003511
```

```
Call:
lm(formula = eblups2 ~ hpdt2, data = datatilsim)

Residuals:
     Min       1Q   Median       3Q      Max
-249.637  -73.450    5.549   73.032  285.217

Coefficients:
              Estimate Std. Error t value Pr(>|t|)
(Intercept)  3.949e-12  1.688e+01   0.000  1.00000
hpdt2       -2.837e+01  8.062e+00  -3.519  0.00096 ***
---
Signif. codes:  0 ?***? 0.001 ?**? 0.01 ?*? 0.05 ?.? 0.1 ? ? 1

Residual standard error: 119.4 on 48 degrees of freedom
Multiple R-squared:  0.2051,Adjusted R-squared:  0.1885
F-statistic: 12.38 on 1 and 48 DF,  p-value: 0.0009596
```

From this output we extract the following parameter values used for subsequent simulation

$$\mu = 4.392 \cdot 10^{-12},$$
$$\beta_1 = -2.424 \cdot 10^{1},$$
$$\beta_2 = 6.210 \cdot 10^{-2},$$
$$\beta_3 = -8.432 \cdot 10^{-1},$$
$$\sigma = 118.2,$$
$$\nu = 3.949e \cdot 10^{-12},$$
$$\beta = -2.837 \cdot 10^{1},$$
$$\sigma_1 = 119.4$$

4 Results

We considered the n ranging from 50 to 250 and calculated power according to the following criteria

- Significant effects of both HPDT and VAS in simulation design (1)
- Significant effect of one or both of HPDT or VAS in simulation design (1)
- Significant effect of HPDT in simulation design (2)

For each n and each criterium we generated 1000 simulated data sets. For criterium 1 and each data set we calculated the p-value as the maximum of two p-values corresponding to adjusted effects of HPDT and VAS, respectively. For criterium 2 the calculated p-value corresponds to an F test for any effect of either HPDT or VAS. For criterium 3 the calculated p-value corresponds to a t-test for effect of HPDT. For each of the criteria the power was calculated as the fraction of times the calculated p-value was below 5%.

From figure 1 and table 1 we se that approximately 170 persons are needed to obtain a power of 80% with criteriumf 1. For the two other criteria powers are well above 80% already with 50 persons.

Number of persons	power (criterium 1)	power (criterium 2)	power (criterium 3)
50	0.195	0.864	0.911
160	0.773	1.000	1.000
170	0.807	1.000	1.000
180	0.851	1.000	1.000

Table 1: Simulated powers to detect an effect of HPDT and VAS for varying number of persons

5 Appendix: R code

```
#power study hyperalgesia 2

#Extracting information from hyperalgesia 1
#For simulation of power in hyperalgesia 2

library(gdata)
hyperdata<-read.xls(file.choose(),sheet=10)
head(hyperdata)
names(hyperdata)
with(hyperdata,length(levels(factor(Forsøgsnummer))))
with(hyperdata,table(X3.Obs..1.A..2.B.))
Day<-rep(1:4,rep(50,4))
personid<-with(hyperdata,rep(Forsøgsnummer,4))
observer<-with(hyperdata,c(X1.Obs..1.A..2.B.,X2.Obs..1.A..2.B.,X3.Obs..1.A..2.B.,X4.Obs..1.A..2.B.))
observer
BTS<-with(hyperdata,c(X1.BTS.Areal..cm2.,X2.BTS.Areal..cm2.,X3.BTS.Areal..cm2.,X4.BTS.Areal..cm2.))
length(BTS)
HPDT<-with(hyperdata,c(X1.HPDT...C.,X2.HPDT...C.,X3.HPDT...C.,X4.HPDT...C.))
MVAS<-with(hyperdata,c(X1.LTS.MAX..mm.,X2.LTS.max..mm.,X3.LTS.max..mm.,X4.LTS.max..mm.))
AVAS<-with(hyperdata,c(X1.LTS.AUC,X2.LTS.AUC,X3.LTS.AUC,X4.LTS.AUC))


datatilsim<-data.frame(personid,Day,observer,BTS,HPDT,MVAS,AVAS)

################################################################
#extracting parameters#########################################
################################################################


modelsim4<-lmer(BTS~1+(1|observer)+(1|personid),data=subset(datatilsim,Day<5))
modelsim3<-lmer(BTS~1+(1|observer)+(1|personid),data=subset(datatilsim,Day<4))
modelsim2<-lmer(BTS~1+(1|observer)+(1|personid),data=subset(datatilsim,Day<3))
summary(modelsim4)
summary(modelsim3)
summary(modelsim2)
```

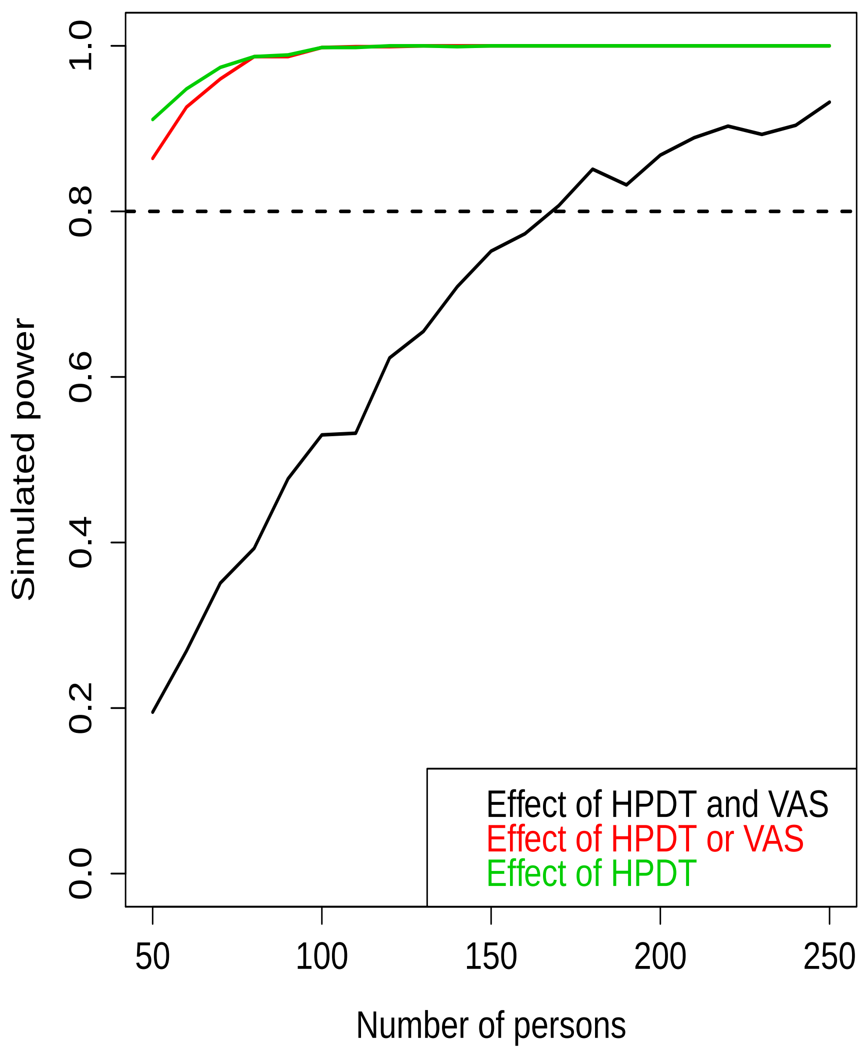

Figure 1: Simulated powers to detect effects of HPDT and VAS for varying number of persons

```
eblups4<-as.vector(unlist(ranef(modelsim4)[[1]]))
eblups3<-as.vector(unlist(ranef(modelsim3)[[1]]))
eblups2<-as.vector(unlist(ranef(modelsim2)[[1]]))



hpdt4<-as.vector(unlist(ranef(lmer(HPDT~1+(1|observer)+(1|personid),data=subset(datatilsim,Day<5)))[[1]]))
hpdt3<-as.vector(unlist(ranef(lmer(HPDT~1+(1|observer)+(1|personid),data=subset(datatilsim,Day<4)))[[1]]))
hpdt2<-as.vector(unlist(ranef(lmer(HPDT~1+(1|observer)+(1|personid),data=subset(datatilsim,Day<3)))[[1]]))

avas4<-as.vector(unlist(ranef(lmer(AVAS~1+(1|observer)+(1|personid),data=subset(datatilsim,Day<5)))[[1]]))
avas3<-as.vector(unlist(ranef(lmer(AVAS~1+(1|observer)+(1|personid),data=subset(datatilsim,Day<4)))[[1]]))
avas2<-as.vector(unlist(ranef(lmer(AVAS~1+(1|observer)+(1|personid),data=subset(datatilsim,Day<3)))[[1]]))

mvas4<-as.vector(unlist(ranef(lmer(MVAS~1+(1|observer)+(1|personid),data=subset(datatilsim,Day<5)))[[1]]))
mvas3<-as.vector(unlist(ranef(lmer(MVAS~1+(1|observer)+(1|personid),data=subset(datatilsim,Day<4)))[[1]]))
mvas2<-as.vector(unlist(ranef(lmer(MVAS~1+(1|observer)+(1|personid),data=subset(datatilsim,Day<3)))[[1]]))

datatilsim<-data.frame(eblups4,eblups3,eblups2,hpdt4,hpdt3,hpdt2,avas4,avas3,avas2,mvas4,mvas3,mvas2)

fit4<-lm(eblups4~hpdt4+avas4+mvas4,data=datatilsim)
fit3<-lm(eblups3~hpdt3+avas3+mvas3,data=datatilsim)
fit2<-lm(eblups2~hpdt2+avas2+mvas2,data=datatilsim)
summary(fit2)

####################################



Call:
  lm(formula = eblups2 ~ hpdt2 + avas2 + mvas2, data = datatilsim)

Residuals:
  Min       1Q   Median       3Q      Max
-236.104  -69.970   -6.324   79.505  308.781

Coefficients:
  Estimate Std. Error t value Pr(>|t|)
(Intercept)  4.392e-12  1.671e+01   0.000  1.00000
hpdt2       -2.424e+01  8.423e+00  -2.878  0.00605 **
  avas2        6.210e-02  5.260e-02   1.180  0.24388
mvas2       -8.432e-01  2.495e+00  -0.338  0.73693
---
  Signif. codes:  0 ?***? 0.001 ?**? 0.01 ?*? 0.05 ?.? 0.1 ? ? 1

Residual standard error: 118.2 on 46 degrees of freedom
Multiple R-squared:  0.2536,  Adjusted R-squared:  0.2049
F-statistic: 5.209 on 3 and 46 DF,  p-value: 0.003511
################################################

baseline.mean<-c(coef(fit2)[1],coef(fit3)[1],coef(fit4)[1])
beta.hpdt<-c(coef(fit2)[2],coef(fit3)[2],coef(fit4)[2])
beta.avas<-c(coef(fit2)[3],coef(fit3)[3],coef(fit4)[3])
beta.mvas<-c(coef(fit2)[4],coef(fit3)[4],coef(fit4)[4])
sd.res<-c(summary(fit2)$sigma,summary(fit3)$sigma,summary(fit4)$sigma)

baseline.mean
beta.hpdt
beta.avas
```

```
beta.mvas
sd.res

########################
> baseline.mean
(Intercept)   (Intercept)   (Intercept)
4.392127e-12  4.957095e-12 -5.164214e-13
> beta.hpdt
hpdt2     hpdt3     hpdt4
-24.24115 -24.53525 -23.40930
> beta.avas
avas2      avas3      avas4
0.06209574 0.03872417 0.03642940
> beta.mvas
mvas2       mvas3       mvas4
-0.84316781  0.06123866 -0.23581709
> sd.res
[1] 118.1687 111.9656 113.7461
>

##############################

fit4.a<-lm(eblups4~hpdt4,data=datatilsim)
fit3.a<-lm(eblups3~hpdt3,data=datatilsim)
fit2.a<-lm(eblups2~hpdt2,data=datatilsim)
summary(fit2.a)

##################

Call:
  lm(formula = eblups2 ~ hpdt2, data = datatilsim)

Residuals:
  Min       1Q   Median       3Q      Max
-249.637  -73.450    5.549   73.032  285.217

Coefficients:
  Estimate Std. Error t value Pr(>|t|)
(Intercept)  3.949e-12  1.688e+01   0.000  1.00000
hpdt2       -2.837e+01  8.062e+00  -3.519  0.00096 ***
  ---
  Signif. codes:  0 ?***? 0.001 ?**? 0.01 ?*? 0.05 ?.? 0.1 ? ? 1

Residual standard error: 119.4 on 48 degrees of freedom
Multiple R-squared:  0.2051,  Adjusted R-squared:  0.1885
F-statistic: 12.38 on 1 and 48 DF,  p-value: 0.0009596

###################

baseline.mean.a<-c(coef(fit2.a)[1],coef(fit3.a)[1],coef(fit4.a)[1])
beta.hpdt.a<-c(coef(fit2.a)[2],coef(fit3.a)[2],coef(fit4.a)[2])
sd.res.a<-c(summary(fit2.a)$sigma,summary(fit3.a)$sigma,summary(fit4.a)$sigma)

baseline.mean.a
beta.hpdt.a
sd.res.a

##############################
> baseline.mean.a
```

```
(Intercept)  (Intercept)  (Intercept)
3.948797e-12 5.996380e-12 6.212621e-13
> beta.hpdt.a
hpdt2     hpdt3     hpdt4
-28.37033 -28.22663 -26.34637
> sd.res.a
[1] 119.3813 112.2607 113.1335
>


###################################
###################################
#Simulation functions##############
###################################
###################################


###################################
#Helping function for sampling from#
#the simultaneous empirical dist of#
#eblups of HPDT,MVAS,AVAS  #########
###################################
empdistmeas<-function(Npers,datatilsim,Ndays){
  n<-dim(datatilsim)[1]
  samples<-sample(1:n,Npers,replace=T)
  personid<-1:Npers
  if(Ndays==2){
    hpdt<-datatilsim$hpdt2
    avas<-datatilsim$avas2
    mvas<-datatilsim$mvas2
  }
  if(Ndays==3){
    hpdt<-datatilsim$hpdt3
    avas<-datatilsim$avas3
    mvas<-datatilsim$mvas3
  }
  if(Ndays==4){
    hpdt<-datatilsim$hpdt4
    avas<-datatilsim$avas4
    mvas<-datatilsim$mvas4
  }
  data.frame(personid,hpdt=hpdt[samples],avas=avas[samples],mvas=mvas[samples])
}

#####################################
#Function for simulating datasets#####
#NB: only one observer here          #
#NBB: number of persons and number of#
#days may be varied##################
#####################################

simulationdata<-function(Ndays,Npers,sd.res,baseline.mean,beta.hpdt,beta.mvas,beta.avas,datatilsim)
{
 sim.emp.data<-empdistmeas(Npers,datatilsim,Ndays)
 hpdt<-sim.emp.data$hpdt
 avas<-sim.emp.data$avas
 mvas<-sim.emp.data$mvas
 if(Ndays==2){
   bts<-baseline.mean[1]+beta.hpdt[1]*hpdt+beta.avas[1]*avas+beta.mvas[1]*mvas+sd.res[1]*rnorm(Npers)
```

```
 }
 if(Ndays==3){
   bts<-baseline.mean[2]+beta.hpdt[2]*hpdt+beta.avas[2]*avas+beta.mvas[2]*mvas+sd.res[2]*rnorm(Npers)
 }
 if(Ndays==4){
   bts<-baseline.mean[3]+beta.hpdt[3]*hpdt+beta.avas[3]*avas+beta.mvas[3]*mvas+sd.res[3]*rnorm(Npers)
 }
   data.frame(bts,hpdt,avas,mvas)
}



simulationdata1<-function(Ndays,Npers,sd.res.a,baseline.mean.a,beta.hpdt.a,datatilsim)
{
  sim.emp.data<-empdistmeas(Npers,datatilsim,Ndays)
  hpdt<-sim.emp.data$hpdt
  if(Ndays==2){
    bts<-baseline.mean.a[1]+beta.hpdt.a[1]*hpdt+sd.res.a[1]*rnorm(Npers)
}

if(Ndays==3){
  bts<-baseline.mean.a[2]+beta.hpdt.a[2]*hpdt+sd.res.a[2]*rnorm(Npers)
}
if(Ndays==4){
  bts<-baseline.mean.a[3]+beta.hpdt.a[3]*hpdt+sd.res.a[3]*rnorm(Npers)
}
  data.frame(bts,hpdt)
}



###############################################
#Simulating power based on 1000 simulations   #
#for each of a number of different scenarios  #
#varying number of persons and number of days #
#with measurements                            #
###############################################

Nsim<-1000
Npers<-rep(seq(50,250,by=10),1)
Ndays<-rep(2,c(21))
power<-rep(0,length(Npers))
power1<-rep(0,length(Npers))
power2<-rep(0,length(Npers))

for (i in 1:length(Npers)){
  count<-0
  count1<-0
  count2<-0
  for (j in 1:Nsim){
    simdat<-simulationdata(Ndays[i],Npers[i],sd.res,baseline.mean.a,beta.hpdt,beta.mvas,beta.avas,datatilsim)
    simdat.a<-simulationdata1(Ndays[i],Npers[i],sd.res.a,baseline.mean.a,beta.hpdt.a,datatilsim)
    fit1<-lm(bts~hpdt+avas+mvas,data=simdat)
    fit21<-lm(bts~hpdt,data=simdat)
    fit22<-lm(bts~avas+mvas,data=simdat)
    fit3<-lm(bts~1,data=simdat)
    fit1.a<-lm(bts~hpdt,data=simdat.a)
    fit2.a<-lm(bts~1,data=simdat.a)
    pval1<-anova(fit1,fit21,test="F")[["Pr(>F)"]][2]
```

```
    pval2<-anova(fit1,fit22,test="F")[["Pr(>F)"]][2]
    pval3<-anova(fit1,fit3,test="F")[["Pr(>F)"]][2]
    pval.a<-anova(fit1.a,fit2.a,test="F")[["Pr(>F)"]][2]
    count<-count+ifelse(pval1<0.05 & pval2<0.05,1,0)
    count1<-count1+ifelse(pval3<0.05,1,0)
    count2<-count2+ifelse(pval.a<0.05,1,0)
  }
  power[i]<-count/Nsim
  power1[i]<-count1/Nsim
  power2[i]<-count2/Nsim
  print(Npers[i])
  print(Ndays[i])
  print(power[i])
  print(power1[i])
  print(power2[i])
}

powerdata<-data.frame(Npers,Ndays,power,power1,power2)
powerdata
###################

Npers Ndays power power1 power2
1     50     2 0.195  0.864  0.911
2     60     2 0.269  0.926  0.948
3     70     2 0.351  0.960  0.974
4     80     2 0.393  0.987  0.987
5     90     2 0.477  0.987  0.989
6    100     2 0.530  0.998  0.998
7    110     2 0.532  0.999  0.998
8    120     2 0.623  0.999  1.000
9    130     2 0.655  1.000  1.000
10   140     2 0.709  1.000  0.999
11   150     2 0.752  1.000  1.000
12   160     2 0.773  1.000  1.000
13   170     2 0.807  1.000  1.000
14   180     2 0.851  1.000  1.000
15   190     2 0.832  1.000  1.000
16   200     2 0.868  1.000  1.000
17   210     2 0.889  1.000  1.000
18   220     2 0.903  1.000  1.000
19   230     2 0.893  1.000  1.000
20   240     2 0.904  1.000  1.000
21   250     2 0.932  1.000  1.000
>


plot(power~Npers,type="l",col=1,lwd=2,data=subset(powerdata,Ndays==2),xlab="Number of persons",ylab="Simulated power",ylim=c(0,1))
with(powerdata,points(y=power1,x=Npers,type="l",col=2,lwd=2))
with(powerdata,points(y=power2,x=Npers,type="l",col=3,lwd=2))
abline(0.8,0,lty=2,lwd=2)
legend("bottomright",
       legend=c("Effect of HPDT and VAS", "Effect of HPDT or VAS", "Effect of HPDT"),
       text.col=1:3)
```
